# Pioneering Enhanced Corrosion Resistance along the Normal Plane of an Ultra-Light Mg-Li Extruded Sheet

**DOI:** 10.3390/ma16196435

**Published:** 2023-09-27

**Authors:** Jiexi Liang, Binbin Deng, Chuanqiang Li, Yong Dong, Naiguang Wang, Zhengrong Zhang, Shidong Wang

**Affiliations:** 1 School of Materials and Energy, Guangdong University of Technology, Guangzhou 510006, China; 2112302170@mail2.gdut.edu.cn (J.L.); 2112102107@mail2.gdut.edu.cn (B.D.); dongyong5205@163.com (Y.D.); wangnaiguang@gdut.edu.cn (N.W.); zzr@gdut.edu.cn (Z.Z.); 2 Key Laboratory of Nuclear Materials and Safety Assessment, Institute of Metal Research, Chinese Academy of Sciences, Shenyang 110016, China; 3 Department of Chemical and Materials Engineering, University of Alberta, Edmonton, AB T6G 2G6, Canada

**Keywords:** magnesium alloy, crystallographic orientation, corrosion anisotropy, corrosion resistance, localized corrosion

## Abstract

The microstructure and corrosion anisotropy of the Mg-5Li extruded sheet were investigated in this work. Three distinct samples cut from the normal plane (A), longitudinal plane (B), and cross-sectional plane (C) of the as-extruded sheet were prepared. The microstructure was analyzed using optical microscopy (OM), scanning electron microscopy (SEM), and X-ray diffraction (XRD). The corrosion resistance and behaviors of the three samples in a 0.1 mol/L NaCl solution were evaluated by employing hydrogen evolution, mass loss testing, electrochemical assessments, and corrosion morphology analyses. The results revealed that sample A displayed a distinctive bimodal (0002) basal texture, along with clearly distinguishably larger grain sizes than the other samples. The effect of grain size and crystallographic orientation on the corrosion resistance was highlighted, indicating the pioneering corrosion resistance of sample A and the lowest corrosion resistance of sample C. Furthermore, all three samples exhibited the characteristic filiform corrosion during the initial stages of corrosion, progressing into the formation of corrosion pits, with sample C displaying pronounced susceptibility.

## 1. Introduction

As an ultra-light magnesium alloy, the density of magnesium–lithium (Mg-Li) alloy is generally 1.35–1.65 g/cm^3^, which is the lightest structural metal material at present. Due to its advantages of high specific strength, good damping, biological compatibility, etc., it is extensively applied in aerospace, electronics, biological industries, and so on [1,2]. However, magnesium generally has a high chemical and electrochemical activity, and lithium possesses a stronger electrochemical activity than magnesium. Adding lithium to magnesium will further destroy the integrity of the magnesium matrix in an aqueous solution, resulting in poorer corrosion resistance than conventional magnesium alloys [3]. Generally, the corrosion resistance of magnesium alloys is affected by their composition and microstructure (such as grain size, intermetallic compounds, and texture) [4,5]. To improve the Mg-Li alloy’s corrosion resistance, adjusting the matrix structure (alloying, second phase, deformation, or heat treatment) and surface modification are commonly used methods [6,7,8,9,10]. After the extrusion deformation of magnesium alloy, the inherent defects, such as the voids and pores of as-cast magnesium alloy, can be eliminated, its mechanical properties can be improved, and its application can be increased. However, the residual impurities and other alloy phases in the manufacturing process of extruded magnesium alloy are prone to result in galvanic corrosion, pitting, and filiform corrosion. The defects leading to the poor corrosion resistance of as-extruded magnesium alloys still exist [11,12]. Previous works have shown that extrusion treatment can change the crystal orientation of magnesium alloy, and the crystal orientation is closely related to the corrosion resistance of magnesium [13,14]. Namely, once the texture is formed in magnesium alloy, corrosion and mechanical anisotropy will occur for the wrought magnesium. Therefore, revealing the corrosion behavior and anisotropy of as-extruded Mg-Li alloys is of great significance for the development of highly corrosion-resistant magnesium alloys. However, there are more reports about the effects of extrusion treatment on the mechanical properties and microstructure of Mg-Li alloys [15,16,17] than on its corrosion anisotropy. The processing indeed affects the crystallographic orientation and mechanical properties of magnesium, resulting in the existence of tension–compression yield asymmetry, the underlying mechanism has been revealed in detail in previous research [18]. However, the addition of lithium can reduce the mechanical anisotropy of commercial magnesium alloys [19]; for instance, the mechanical anisotropy of as-extruded AZ31 alloy can be remarkably altered with the addition of lithium, and the AZ31-5 wt.% Li alloy presents the weakest planar anisotropy [19]. Lithium additions can generally reduce the c/a ratio and refine the recrystallized structure, leading to the rotation of basal poles in the transverse direction [19]. Even though the crystallographic orientation and mechanical anisotropy of Mg-Li alloys seem to be clarified at present, the effect of modifying crystallographic orientation induced by lithium addition on the corrosion resistance of Mg-Li alloy is hardly reported.

In this work, different from the study on the mechanical anisotropy and corrosion behavior of conventional magnesium, an ultra-light Mg-Li extruded sheet is selected to reveal the effect of crystallographic orientation on its corrosion resistance, confirming the pioneering enhanced corrosion resistance along the normal plane of the extruded sheet. Hydrogen evolution, weight loss, electrochemical test, and corrosion morphology observation were carried out on three differently orientated samples to achieve the aims of this work, i.e., to discover the microstructure, corrosion anisotropy, and behaviors of as-extruded Mg-5Li binary alloy. Only two elements, Mg and Li, are contained in the Mg-5Li alloy, and only a single α-Mg matrix phase can be formed when the Li content is less than 5.7 wt.% based on the Mg-Li binary phase diagram [20]; therefore, there is no effect of the particles in the structure of the alloy on the corrosion resistance. As a result, the different corrosion properties of three different sections in as-extruded Mg-5Li binary alloy were analyzed, and the microstructure and corrosion anisotropy of the alloy were also revealed in detail.

## 2. Materials and Methods

### 2.1. Materials Preparation and Microstructural Analysis

**Figure 1:**
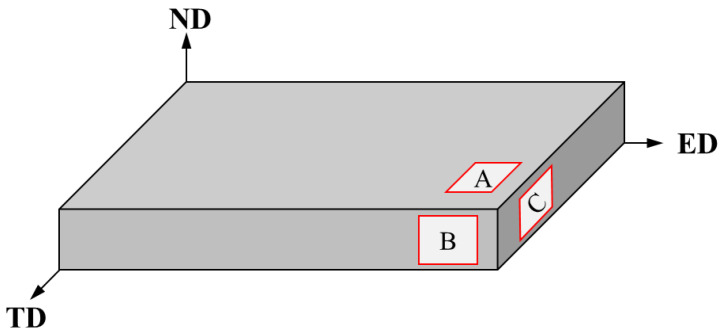
Schematic diagram of samples A, B and C cut from as-extruded Mg-5Li alloy.

The experimental raw materials are pure magnesium ingot (99.9%) and lithium ingot (99.9%), and the preparation process is as follows: the high-purity magnesium ingot is melted through the resistance furnace, and then the mass fraction of 5 wt.% lithium is added under the conditions of continuous passage of SF_6_ and CO_2_ protective gas, and fully stirred. After the temperature drops to 730 °C, LiCl and LiF covering agents are added in the process, and CO_2_ or air and SF_6_ mixed gas are injected to protect the alloy from melting. After maintaining the temperature of the alloy at 710 °C for 10–20 min, the alloy is poured out of the square copper mold and cooled to obtain square ingots. Then, the ingot is extruded into a plate with a cross-sectional size of 12 × 50 mm at an extrusion temperature of about 300 °C, with an extrusion ratio of about 6.5. The samples cut from the extruded plates were ground with SiC papers up to 5000 grits, and finally finely polished up to a 1μm finish with ethanol. All the samples keep the same surface treatment for the same testing, indicating the trivial effect of surface roughness on the corrosion resistance of different samples. The schematic diagram of three differently orientated samples is shown in Figure 1, and the samples are marked as sample A(ED-TD), sample B (ED-ND), and sample C (TD-ND), respectively. After grinding and polishing, the samples were etched with 5% nitrate alcohol solution for about 16 s to observe the microstructure using scanning electron microscope (SEM) and optical microscope (OM). In addition, sample A was analyzed using Bruke D8 ADVANCE X-ray diffractometer (Bruker, Karlsruhe, Germany) to determine the crystal orientation characteristics of the alloy.

### 2.2. Hydrogen Evolution, Weight Loss, and Immersion Testing

For hydrogen evolution and weight loss experiments, the sample was mounted with epoxy resin to ensure that only one surface A\B\C was exposed. Then, the sample with a polished surface was soaked in 0.1 mol/L NaCl solution for three days. During the immersion, hydrogen gas was collected using an inverted funnel and burette above the immersed samples to achieve the hydrogen evolution rate. Following the immersion exposure period, all the samples were cleaned in a chromic acid solution of 180 g/L chromium trioxide to remove any surface products, and thus the weight loss rate of different samples could be calculated. The samples were weighed using a high-precision scale with a minimum accuracy of 0.1 mg to guarantee accuracy. As for the corrosion morphologies, OM and SEM techniques were used to observe the corrosion evolution of samples A\B\C in 0.1 mol/L NaCl solution. In addition, the corrosion depth of three samples was also observed using confocal laser scanning microscopy (CLSM, OLYMPUS LEXT OLS4100, Olympus, Tokyo, Japan).

### 2.3. Electrochemical Testing

Potentiodynamic polarization measurements were carried out with a scan rate of 1 mV/s from −1.8 V_SCE_ to −1.1 V_SCE_ by using a Wuhan CorrTest electrochemistry test system (CS350H, Wuhan, China) at room temperature. The three-electrode system was used: working electrode (sample), counter electrode (platinum electrode), and reference electrode (saturated calomel electrode). OCP testing was performed for 10 min before the polarization to ensure the stability of the electrochemical system test. The potentiodynamic polarization curves of differently orientated samples of the alloy were compared and analyzed. To ensure reliability, at least three samples of each state were examined to determine the corrosion parameters.

## 3. Results and Discussion

### 3.1. Microstructure

**Figure 2:**
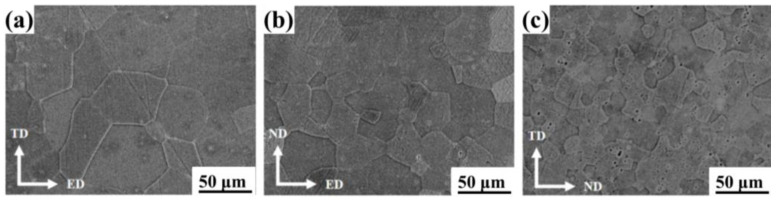
SEM images of samples (**a**) A, (**b**) B, and (**c**) C cut from the as-extruded Mg-5Li alloy.

**Figure 3:**
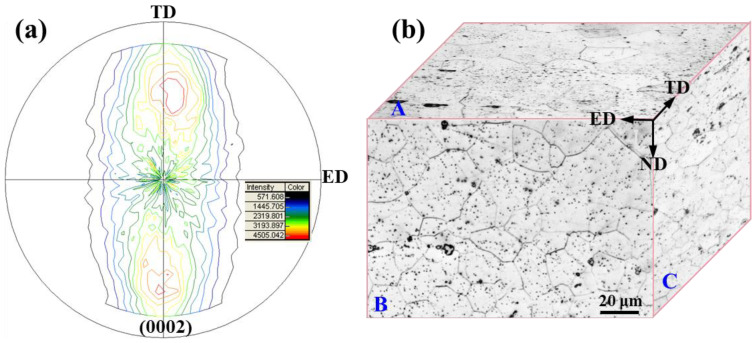
(**a**) (0002) pole figure of sample A cut from the as-extruded Mg-5Li alloy and (**b**) 3D OM images.

The microstructure of three differently orientated samples, A, B, and C, cut from as-extruded Mg-5Li alloy are displayed in Figure 2. It can be seen that the deformation characteristics of the three samples are not obvious after extrusion deformation, and all of them show the equiaxed grain structure. The average grain size of A, B, and C is about 50 ± 5 μm, 30 ± 4 μm and 20 ± 4 μm, respectively. The texture is generally formed by the deforming Mg alloy. To further characterize the texture characteristics of as-extruded Mg-5Li alloy, the XRD texture testing was conducted on sample A, and the results are shown in Figure 3a. Meanwhile, the OM microstructure in three directions of as-extruded Mg-5Li alloy is also displayed in Figure 3b, and is consistent with the SEM results in Figure 2. In general, the strong (0002) basal texture is prone to be formed by the Mg alloy during hot extrusion deformation, and the (0002) plane is parallel to the extrusion direction (ED) [21]. In this work, the as-extruded Mg-5Li alloy exhibits a bimodal (0002) basal texture, that is, the (0002) plane is parallel to the extrusion direction ED, and at the same time it tilts about 60° in the TD direction, which is different from the as-extruded fiber texture of conventional Mg alloys. Due to the addition of lithium, the c/a ratio of the Mg alloy is reduced, and thus the (0002) basal plane tilts in the direction of TD [22]. Meanwhile, it can be speculated that the difference in grain orientation between samples A and B should be trivial, and also the distribution of differently orientated grains in the two samples is relatively random, which is consistent with the previous work [23]. However, the grain orientation of sample C is very different from those of samples A and B, that is, sample C mainly presents prismatic texture characteristics. In general, there is some connection between the crystal orientation and corrosion resistance of the Mg alloy [24,25]. Li et al. studied the influence of different lithium contents on the corrosion behavior of as-extruded Mg-Li alloys and found that the corrosion resistance of binary Mg-5Li alloy with relatively random grain orientation is significantly better than that of Mg-1Li alloy, with preferred grain orientation [23].

### 3.2. Corrosion Resistance

**Figure 4:**
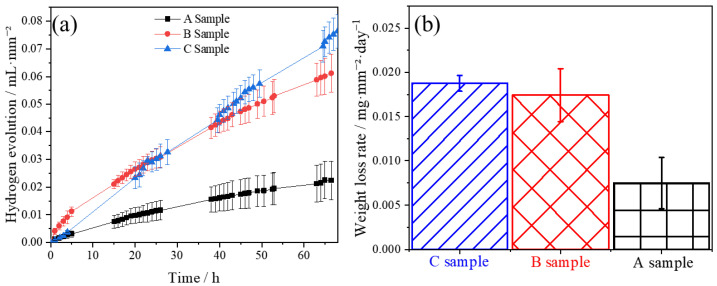
(**a**) Hydrogen evolution and (**b**) weight loss of samples A, B and C of as-extruded Mg-5Li alloy.

The hydrogen evolution and weight loss of samples A, B, and C are displayed in Figure 4. It can be seen that the hydrogen evolution rate of sample A is the smallest, while the hydrogen evolution rate of sample B is the fastest at the early corrosion stage (Figure 4a). However, the rate of hydrogen evolution for sample C surpasses sample B after immersion for 24 h, but the upward trend of hydrogen evolution for the two samples is similar after long-term corrosion. However, the hydrogen evolution of sample A is always the slowest, which indicates that sample A has the best corrosion performance, i.e., the ND plane of as-extruded Mg-5Li alloy is most corrosion resistant. The compared weight loss rates of the three samples confirm the rationality of the aforementioned hydrogen evolution. Therefore, sample A of the as-extruded Mg-5Li alloy presents the highest corrosion resistance, while B and C planes exhibit similar but lower corrosion resistance. Previous works have revealed that coarse grains can form an effective corrosion barrier to slow down the intergranular corrosion (IGC), resulting in the high corrosion resistance of magnesium, while fine-grained samples possess a higher negative potential, more chemical activity, and a faster corrosion rate [26,27]. In addition, grain refinement may lead to the increase in grain boundaries, and the high density of grain boundaries indicates the high fraction of dislocation defects, resulting in a higher corrosion damage at boundaries and thus accelerating the corrosion propagation [27]. Therefore, sample A, with the largest grain size (Figure 2), presents the highest corrosion resistance, while sample C, with the smallest grain size, corresponds to the worst corrosion resistance. However, the grain size of sample B is in the middle, but the corrosion resistance is similar to that of sample C, which is likely closely related to the texture characteristics. Based on the texture analysis in Figure 3, it can be deduced that sample C mainly exposes the prismatic plane, while sample B presents both the base and prismatic plane. Previous works have demonstrated that the corrosion resistance of the basal plane is higher than that of the prismatic plane in HCP Mg alloys due to the galvanic corrosion effect between the cathodic base plane and the anodic prismatic plane [13]. Therefore, although the grain size of sample B in this study is medium, the micro-galvanic effect between the base plane and prismatic plane accelerates the corrosion, so that the improving corrosion resistance arising from the larger grain size is discounted by the above-mentioned micro-galvanic corrosion effect. As a result, both samples B and C present a similar corrosion resistance. On the other hand, for sample A, although micro-galvanic corrosion also took place, the contribution of grain size to improving corrosion resistance is dominant, resulting in the best corrosion resistance.

**Figure 5:**
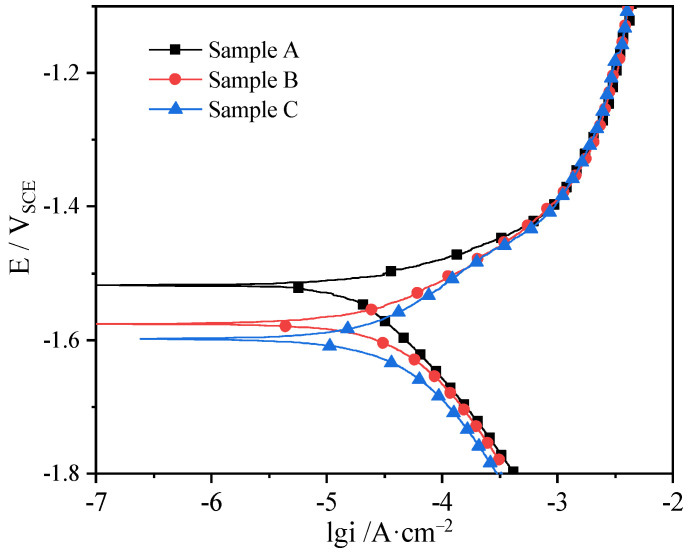
Polarization curves of samples A, B and C of as-extruded Mg-5Li alloy.

Figure 5 indicates the potentiodynamic polarization curves of samples A, B, and C of as-extruded Mg-5Li alloy. It can be seen that none of the three anodes present the passivation behavior, indicating that an effective protective film cannot be formed on the surface, which conforms to the corrosion feature of Mg alloy. From the cathode reaction kinetics and corrosion current density, it can be seen that there is no apparent difference for the three samples, indicating that the corrosion mechanism of this alloy is dominated by anodic dissolution. On the other hand, the self-corrosion potential (E_corr_) of the three samples follows the order of E_corr-A_ (−1.52 ± 0.02 V_SCE_) > E_corr-B_ (−1.58 ± 0.03 V_SCE_) > E_corr-C_ (−1.60 ± 0.02 V_SCE_). In general, the higher the self-corrosion potential of Mg alloys, the better the corrosion resistance. Therefore, the corrosion resistance of sample A is higher than that of samples B and C, corresponding to the results of hydrogen evolution and weight loss rate in Figure 4. In addition, previous work has reported that the Mg-3Al-5Pb-1Ga-Y alloy with a smaller grain size exhibited a greater negative potential, higher chemical activity, and faster corrosion rate [27], which also confirms the rationalization of the results achieved in this work. Therefore, the corrosion resistance of as-extruded Mg-5Li alloy is different in different sections, i.e., the corrosion resistance is the lowest on C’s surface (cross-section), the largest on A’s surface (normal section), and in the middle on B’s surface (longitudinal section).

### 3.3. Corrosion Morphologies

**Figure 6:**
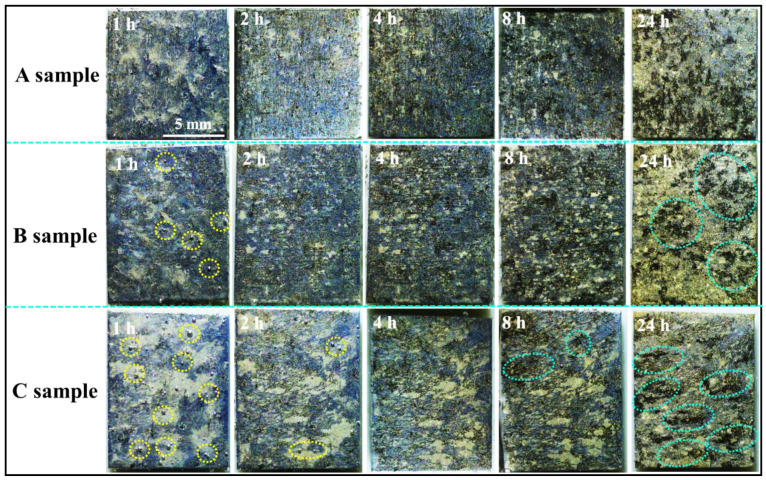
In situ corrosion morphologies of samples A, B and C of as-extruded Mg-5Li alloy after immersion for different time in 0.1 mol/L NaCl solution. The yellow circles indicate the original sites of hydrogen generation, and the green circles point out the large corrosion pits.

**Figure 7:**
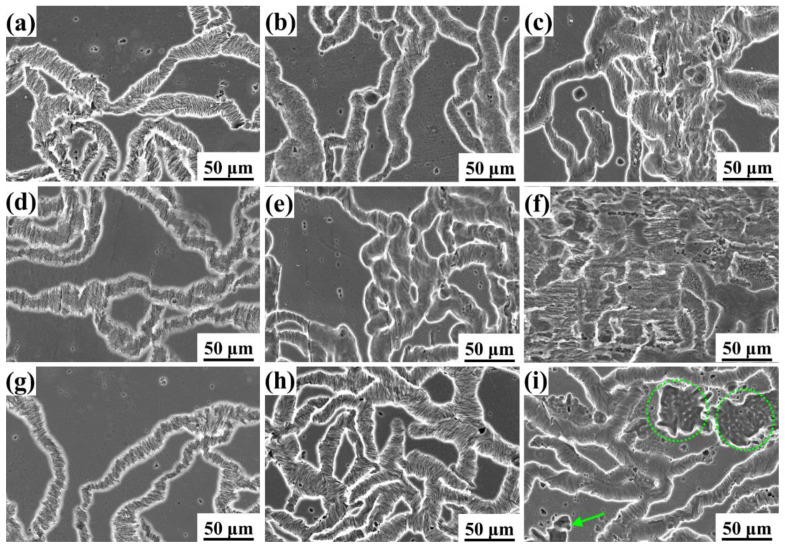
SEM observation of (**a**–**c**) sample A, (**d**–**f**) sample B, and (**g**–**i**) sample C of as-extruded Mg-5Li alloy after immersion for (**a**,**d**,**g**) 2 h, (**b**,**e**,**h**) 8 h, and (**c**,**f**,**i**) 24 h in 0.1 mol/L NaCl solution. The circles and arrows in image (**i**) indicate the large corrosion pits.

Figure 6 shows the in situ OM corrosion morphology evolution of three samples of as-extruded Mg-5Li alloy soaked in 0.1 mol/L NaCl solution for different times. It can be obviously observed that many of hydrogen bubbles are quickly formed on the local surface of sample C (as marked by yellow dotted circles in Figure 6); sample B presents a moderate number of hydrogen bubbles, whilst the number of hydrogen bubbles on the surface of sample A is trivial in the initial immersion period. With the increasing immersion time, severe local corrosion occurs for sample C after 8 h of immersion (as marked by green dotted ellipses in Figure 6), while sample A presents a relatively uniform corrosion, and sample B is still in the middle. On the other hand, even though a much more intact surface can be observed for sample C at the initial corrosion, the corrosion depth is the largest, resulting in the worst surface condition after a long-term immersion, which can be confirmed by the following SEM and confocal laser scanning microscopy. The SEM observation of corrosion morphologies of different samples soaking for different times is shown in Figure 7, revealing that the typical filiform corrosion occurs for the three samples of as-extruded Mg-5Li alloy, and the corrosion filaments gradually propagate to cover almost the whole surface with the increase in soaking time. In general, the filiform corrosion of magnesium is related to the reported dynamic evolution of local anode and cathode positions [28,29], and the proceeding corrosion filaments also present an orientation for the wrought magnesium due to the existence of crystal texture and/or the change of grain boundary and twin boundary orientation [30,31]. In fact, the corrosion filaments propagate randomly for all three samples and no obvious orientation can be observed in this work, which can be attributed to the often-weak texture of Mg-Li alloys compared with other conventional Mg alloys. It should be noted that the tiny corrosion filaments at the bottom of corrosion grooves are apparent for the three samples after 2 h of immersion, and this feature remained for sample C until 8 h of immersion; however, the bottom of the corrosion grooves for sample A become blunt after 8 h of immersion (Figure 7b). Based on the texture analysis (Figure 3), the prismatic planes exposed to the solution for sample C exhibit much higher electrochemical activity than the massive base planes of sample A. As a result, the corrosion resistance of sample C is lower than that of sample A, which is consistent with the previous work reporting that the corrosion resistance of the basal plane of magnesium is higher than that of the prismatic plane [13]. After immersion for 24 h, large corrosion pits begin to occur in sample C (marked in Figure 7i), and sample B exhibits massive corrosion damage.

**Figure 8:**
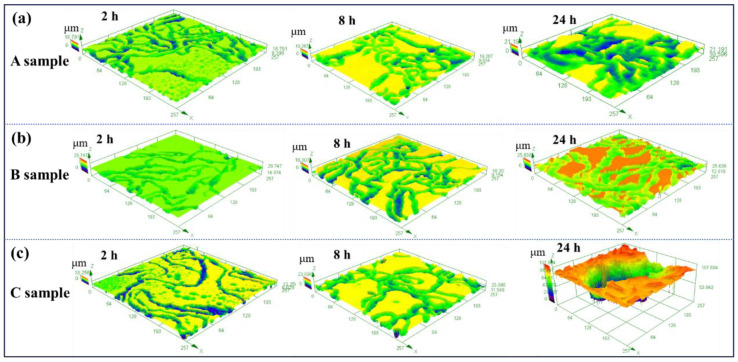
Representative 3D CLSM images of (**a**) sample A, (**b**) sample B, and (**c**) sample C after immersion in 0.1 M NaCl solution for 2 h, 8 h and 24 h.

When HCP Mg-Li alloy is soaked in NaCl solution, loose and porous Mg(OH)_2_ will be formed on the surface. The existence of these surface oxide films can slow down the corrosion of the alloy to a certain extent, but these porous oxide films in NaCl solution are also an important reason for the formation of filiform corrosion. Chloride ions in the solution are enriched at the head of the corroded wire, leading to the corrosion expanding in the horizontal direction and forming filamentary corrosion [32,33]. In addition, the chloride ions will accumulate in the anode region, and the difference in the concentration of chloride ions in the etched filament head and the etched filament tail is the driving force of the etched filament’s propagation [34]. In this work, the filamentous corrosion on the three samples is obvious, and they also suffer pitting corrosion after long-term corrosion, especially for sample C. In addition to comparing the corrosion morphologies in the plane for the three samples, the corrosion depth is also observed, and the results are displayed in Figure 8. For sample A, the corrosion depth changes slightly, but the corroded area is increased with the increase in soaking time. The counterparts for sample B present an accelerating trend both in corrosion depth and areas at the same immersion time. However, for sample C, the variation in corrosion depth dramatically increases with the immersion time, even though the corrosion areas are similar to the other two samples. Therefore, the corrosion morphologies herein demonstrate that the corrosion resistance of sample C is the worst after a long-term immersion in 0.1 mol/L NaCl solution.

Based on the findings in this work, if the application of an as-extruded Mg-Li sheet is carried out in practice, for example, in electronics, automotive, aerospace, etc., fields, it offers great potential for vehicle lightweighting. The preferential surface choice exposed to the environment for the as-extruded Mg-Li sheet should be the normal plane due to the pioneering enhanced corrosion resistance along this plane when compared with the other two planes. As a result, the novel findings herein provide guidance in the design and application of Mg-Li alloys in the future.

## 4. Conclusions

Through revealing the relationship between the crystallographic texture and corrosion resistance of the Mg-5Li extruded sheet, the following conclusions can be drawn:

(1) The corrosion resistance of different sectional samples cut from as-extruded Mg-5Li alloy reduces in the order of sample A (ND plane) > sample B (ED plane) > sample C (TD plane), primarily due to the various effects of texture and grain size.
(2) All the different sectional samples in as-extruded Mg-5Li alloys exhibit the typical filiform corrosion at the initial corrosion stage, but the large corrosion pits gradually emerge, prolonging the corrosion time, with sample C displaying pronounced susceptibility.
(3) Corrosion anisotropy indeed exists in the as-extruded Mg-5Li alloy testing in 0.1 mol/L NaCl solution, indicating the best corrosion performance of the normal plane and itsl possible application in practice.

## Data Availability

The data presented in this study are available upon request from the corresponding author.
